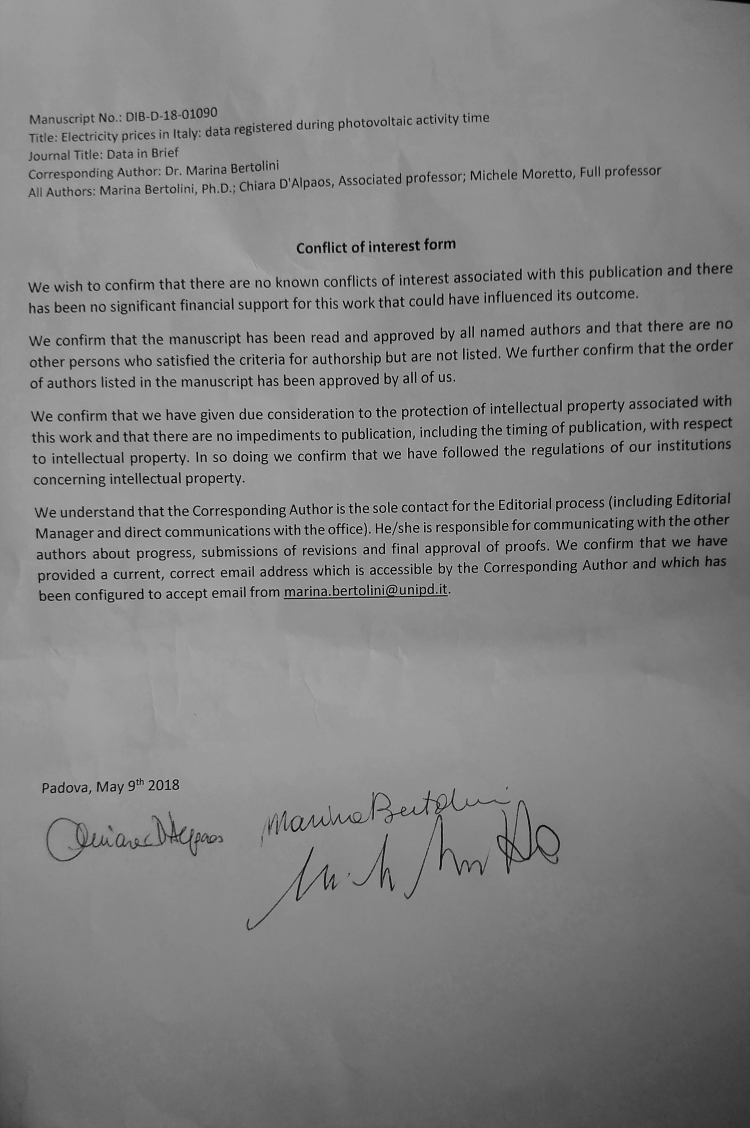# Electricity prices in Italy: Data registered during photovoltaic activity interval

**DOI:** 10.1016/j.dib.2018.06.018

**Published:** 2018-06-19

**Authors:** Marina Bertolini, Chiara D’Alpaos, Michele Moretto

**Affiliations:** a Department of Economics and Management, University of Padova, CRIEP and Centro Studi “Giorgio Levi Cases”, via del Santo, 33, Padova, Italy; b Department of Civil, Architectural and Environmental Engineering, University of Padova and Centro Studi “Giorgio Levi Cases”, Padova, Italy; c Department of Economics and Management, University of Padova, Fondazione Eni Enrico Mattei (FEEM) Centro Studi “Giorgio Levi Cases”, Padova, Italy

**Keywords:** PV prices, Electricity prices, Electricity markets

## Abstract

Data we present in this article are related to the research article by Bertolini M., D’Alpaos C. and Moretto M. “Do Smart Grids boost investments in domestic PV plants? Evidence from the Italian electricity market”(Bertolini et al., 2018) [1]. The dataset is an elaboration of historical spot prices provided by the Italian Gestore dei Mercati Energetici (GME) and gives information about average prices registered during the day, when photovoltaic (PV) plants are – on average – expected to produce energy. Prices are stated in €/MWh.

**Specifications Table**

**Table:** 

Subject area	*Economics*
More specific subject area	*Finance, project evaluation*
Type of data	*Table and figure*
How data was acquired	*Download from the database provided by the Gestore dei Mercati Elettrici (GME)*
Data format	*Elaboration*
Experimental factors	*N.A.*
Experimental features	*N.A.*
Data source location	*Italian electricity spot market, by geographical zones*
Data accessibility	*Data are attached with this article and are available in raw version at*http://www.mercatoelettrico.org/It/Statistiche/ME/DatiSintesi.aspx

**Value of the data**

• Differently from raw data on electricity market prices, the dataset provides information about prices registered in a specific time-slot, giving a synthesis of market results during the PV activity. In this way, the dataset helps to study the effect of PV presence on the electricity markets.
• Time series have been built for six geographical areas, corresponding to the Italian price zones: further researches may study differences among zones for what regards PV penetration, e.g. considering power installed and plants set on the areas.
• The dataset can be used for comparisons among countries while looking at the PV effect on electricity markets.

## 1 Data

The dataset contains daily electricity prices registered in Italy during the hours in which photovoltaic plants are supposed to be – on average – active. Time series cover a period of seven years between 2010 and 2016, and they are provided for the six geographical zones in which the Italian electricity market is organized.

While studying interactions between PV plants and the electricity markets, it is important to consider the time interval in which the plants are effectively active: this is particularly true for analysis performed in absence of storage [1]. This interval depends on a number of plant-specific characteristics, such as latitude and inclination [2]. Seasons and weather conditions heavily change production patterns; effective production depends on a number of factors, also depending on Operations and Maintenance activity, i.e. the presence of dust on panels [3], but the effect on prices of these production distortions is negligible. For sake of simplicity, evaluation models can look at a standard interval. In building the dataset, we considered the standard interval of prices registered between 8 a.m. and 8 p.m., corresponding to the time slot “F1”, determined by the Authority (ARERA – Regulation Authority for Energy, Gas and Water, named AEEG – Authority for Electricity and Gas at the time of the decree of our interest) with the decree 181/06 [4]. The dataset origins from the Company managing the Energy Markets (GME - Gestore dei Mercati Energetici) dataset [5] on electricity prices registered on the spot market (day-head market).

The time series cover the period between 2010 and 2016, which was the latest completed year when we performed the analysis in Ref. [1]. The dataset starts from 2010 because in that period the number of PV plants entering the market was very high [6], thanks to the intense incentive program developed by the Italian government [7]. Data from 2010 incorporate the effect of PV plants’ presence in the market, and this make the time interval suitable for analysis for this particular market.

Time series are provided for six price zones, corresponding to six Italian geographical areas in which the market is divided [8]:

– Northern Italy (GME label: NORD), that comprehends Val D’Aosta, Piemonte, Liguria, Lombardia, Trentino, Veneto, Friuli Venezia Giulia and Emilia Romagna region;
– Central-Northern Italy (GME label: CNORD) that includes Toscana, Umbria and Marche region;
– Central-Southern Italy (GME label: CSUD) corresponding to Lazio, Abruzzo and Campania;
– Southern Italy (GME label: SUD) including Molise, Puglia, Basilicata and Calabria region;
– Sicily (GME label: SICI);
– Sardinia (GME label: SARD).

The series we present in this article consist in the average daily price registered in the six zones, stated in €/MWh.

## 2 Experimental design, materials and methods

In the original dataset, observations on prices are provided for each hour of the day, for each day of the year. In our elaboration, we collapsed hourly data in daily data, by calculating a simple mean price between 8.00 a.m. and 8.00 p.m. (mean of 12 spot prices per day). As discussed in the previous paragraph, this interval can be considered the interval in which PV plants are producing energy, thus having an impact on electricity prices.

**Fig. 1:**
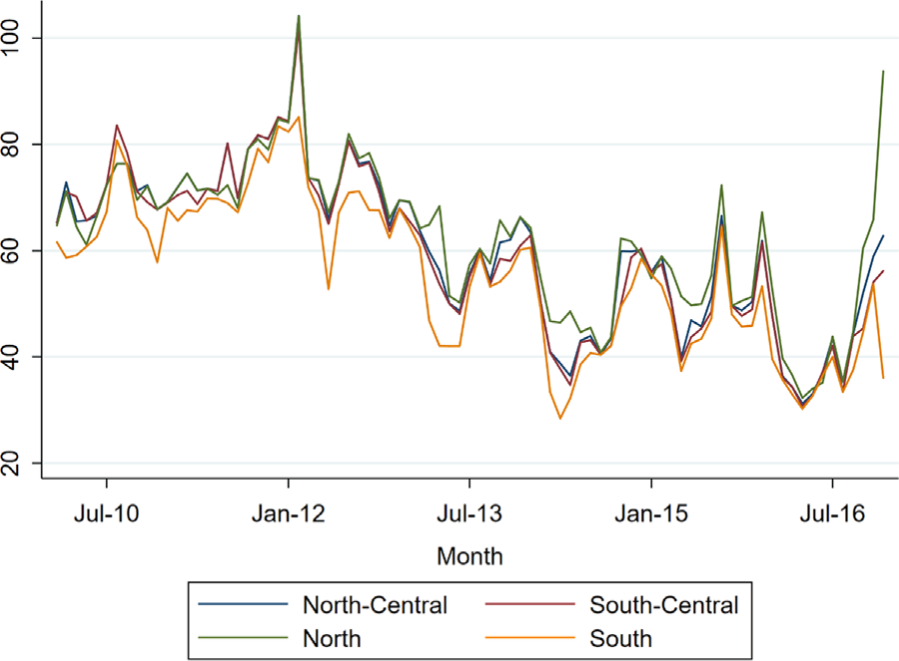
monthly data for the 4 areas analyzed in Ref. [1].

Further analysis of the dataset can be carried out by making a second collapse of daily averages in monthly averages (Fig. 1). It is possible to demonstrate that these new series, opportunely elaborated (i.e. avoiding seasonality issues), behave like a Geometric Brownian Motion [1]: this conclusion do not apply to Sicily and Sardinia regions, whose prices are heavily affected by the island condition.

## Figures and Tables

**Fig. S1:**